# Transcriptomic Analysis Reveals Regulatory Responses of Fatty Acid Positional Distribution in Triacylglycerols and Lipid Composition to Dietary n-3 HUFA in the Muscle of *Trachinotus ovatus*

**DOI:** 10.3390/ani15162427

**Published:** 2025-08-19

**Authors:** Xin Gao, Mengmeng Li, Junfeng Guan, Zhiyi Cheng, Dizhi Xie, Yuanyou Li

**Affiliations:** 1 College of Marine Sciences, South China Agricultural University, Guangzhou 510642, China; xingao@stu.scau.edu.cn (X.G.); a374168380@163.com (J.G.); 13220224680@163.com (Z.C.); 2 College of Agronomy, Liaocheng University, Liaocheng 252059, China; mmli@lcu.edu.cn

**Keywords:** lipid composition, muscle, phospholipids, positional distribution of fatty acids, *Trachinotus ovatus*

## Abstract

The nutritional value of lipids depends not only on their fatty acid composition but also on their stereospecific positioning on the glycerol backbone. This study investigated the fatty acid composition and *sn*-2 positional distribution of triacylglycerols (TAG), as well as the composition of major phospholipids in golden pompano (*Trachinotus ovatus*) juveniles (initial weight: 10 g) fed five diets including graded levels of dietary n-3 highly unsaturated fatty acids (HUFA; 0.64–2.10%) for 56 days. With increasing dietary n-3 HUFA levels, the proportions of eicosapentaenoic acid (EPA), docosapentaenoic acid (DPA), docosahexaenoic acid (DHA), and total n-3 HUFA in muscle TAG, phosphatidylcholine (PC), and phosphatidylethanolamine (PE) significantly increased. Phospholipids, especially PC and PE, were preferentially enriched with n-3 HUFA, and the *sn*-2 positions of TAG showed a significantly increased deposition of DHA and reduced n-6/n-3 ratios. RNA-Seq analysis was performed on muscle tissues of *T. ovatus* subjected to different dietary n-3 HUFA levels to further investigate the molecular mechanisms of lipid compositional and structural changes. A total of 126,792 unigenes were obtained, of which 47.78% were successfully annotated. KEGG pathway enrichment analysis implicated the glycerophospholipid, glycerolipid, and sphingolipid metabolism pathways in lipid composition and distribution regulation, identifying *gpat4*, *agpat3*, *agpat8*, *lpeat1*, and *lpgat1* as potential regulators. These findings offer insights into lipid remodeling in marine fish and support strategies to enhance aquaculture product quality.

## Simple Summary

This study examined how different levels of dietary n-3 highly unsaturated fatty acids (HUFA) affect the fatty acid composition and positioning in the muscle lipids of golden pompano (*Trachinotus ovatus*). Higher dietary n-3 HUFA increased beneficial fatty acids like EPA and DHA, especially in phospholipids and in specific positions on triglycerides. Gene expression analysis identified key genes involved in lipid metabolism. These results help explain how diet influences fish muscle lipids and can guide feed formulators in improving aquaculture product quality.

## 1. Introduction

Fish, especially marine species, constitute a major natural source of highly unsaturated fatty acids (HUFA) such as eicosapentaenate (EPA; 20:5n-3) and docosahexaenoate (DHA; 22:6n-3), which are beneficial for human health, exhibiting anti-inflammatory effects and reducing the risk of cancer and cardiovascular disease [1,2]. In teleost fish, HUFA also plays important roles in maintaining individual survival, growth, development, and reproduction [3,4]. HUFA—particularly DHA—is predominantly incorporated into membrane phospholipids (PL) or stored in triacylglycerols (TAG) and is rarely used for oxidative energy production. This suggests that HUFA is primarily utilized for deposition rather than catabolism in fish muscle. Previous studies have shown that dietary lipid sources significantly affect HUFA content in both the TAG and PL fractions of *Oreochromis niloticus*, while exerting little influence on its positional distribution within the glycerol backbone [5]. Similar findings have been reported in *Salmo salar* [6], further supporting the notion that dietary composition plays a critical role in shaping the structure of muscle lipids in fish.

Emerging evidence suggests that lipid molecular structure, particularly fatty acid regiospecificity on the glycerol backbone, significantly influences nutritional value [7]. EPA and DHA at the *sn*-2 positions of TAG in fish oil (FO) have been shown to exhibit greater bioavailability compared to those at the *sn*-1/3 positions [8]. In mice, DHA at *sn*-2 in TAG significantly reduces serum cholesterol and TAG levels [9]. In addition to regiospecificity, lipid class turnover also influences the nutritional value of fish flesh [10]. Fish muscle lipids primarily consist of TAG, phosphatidylcholine (PC), and phosphatidylethanolamine (PE), with TAG comprising over 70% of total lipids [11]. HUFA is extremely susceptible to peroxidation because of their high degree of unsaturation, while at the *sn*-2 postion of phospholipids, it can be protected against oxidative damage [12].

Changes in lipid composition represent a dynamic mechanism vital for maintaining membrane structure and lipid balance [13,14]. At the molecular level, this process primarily involves two pathways, i.e., the glycerolipid and glycerophospholipid metabolic pathways [15]. Glycerolipid remodeling occurs through two primary mechanisms: (1) acyl chain modification and (2) head group substitution. The acyl chain remodeling process involves the exchange of one or both fatty acid moieties via a lysophospholipid (LPL) intermediate. In animal systems, this phospholipid acyl remodeling is known as the Lands cycle [14], in which fatty acids attached to the *sn*-2 position of PL are liberated by phospholipases. The resultant LPLs can be reacylated by lysophospholipid acyltransferases (LPLATs) to generate PL with different fatty acids. Alternatively, processes of acyl remodeling can include an exchange of both acyl groups with a glycerophosphodiester (GPD) or diacylglycerol (DAG) intermediate. Key enzymes such as glycerol-3-phosphate acyltransferase (GPAT), 1-acylglycerol-3-phosphate O-acyltransferase (AGPAT), lysophosphatidylethanolamine acyltransferase (LPEAT), and lysophosphatidylglycerol acyltransferase (LPGAT) play essential roles by catalyzing the stepwise esterification of fatty acids to the glycerol backbone, thereby determining the composition and positional distribution of fatty acids in TAG and PL [13,15]. Studies in fish have demonstrated that dietary lipid sources not only alter tissue lipid classes and fatty acid composition, but also influence the positional distribution of EPA and DHA within lipid molecules, as observed in species such as *S. salar* [6,16], *Oncorhynchus tshawytscha* [17], and *Scomber australasicus* [18]. However, the regulatory mechanisms controlling the transcript levels and activities of enzymes involved in membrane lipid remodeling remain to be elucidated. Given this, we hypothesize that dietary HUFA may alter fatty acid distribution (particularly at the *sn*-2 position), thereby enhancing the nutritional value of edible fish portions through improved bioavailability of n-3 fatty acids.

Golden pompano (*Trachinotus ovatus*) is one of the most economically important marine carnivorous species cultured in South China [19]. Due to its rapid growth rate, short reproductive cycle, favorable organoleptic properties, and high nutritional value, *T. ovatus* has garnered considerable attention from both consumers and aquaculture producers. Despite its commercial significance, the molecular mechanisms underlying lipid metabolism and fatty acid composition in *T. ovatus* muscle remain poorly understood. The present study conducted an eight-week feeding trial utilizing diets with varying levels of n-3 HUFA. Muscle lipid composition and structural characteristics were analyzed, and RNA-Seq was used to identify key genes and pathways involved in lipid composition remodeling and fatty acid positional distribution. The aim of this study is to provide a comprehensive understanding of how dietary HUFA regulates lipid molecular structure in marine fish muscle and to offer molecular insights for improving nutritional quality through feed formulation.

## 2. Materials and Methods

### 2.1. Fish, Diet Formulation, Feeding, and Sample Collection

*Trachinotus ovatus* juveniles (initial mean weight: 10 g) were purchased from a hatchery in Guangdong Province. A total of 375 fish were randomly distributed into 15 net cages (1.0 m × 1.0 m × 1.5 m) at a rate of 25 fish per cage. The fish were fed a mixture containing equal amounts of the five experimental diets. Fish were reared in floating net cages and fed five isonitrogenous diets with graded n-3 HUFA levels (0.64%, 1.00%, 1.24%, 1.73%, and 2.10%) for 56 days. Then, fish were hand-fed to apparent satiation twice a day (5:30 and 17:30) for 8 weeks. Water temperature ranged from 25 to 30 °C, with salinity from 30 to 33 ppt, ammonia nitrogen < 0.5 mg L^−1^, dissolved oxygen > 5.0 mg L^−1^, and a natural daylight cycle.

At the end of the experiment, fish were anesthetized with MS-222 (100 mg/mL), and six fish from each cage were randomly selected. Muscle samples were collected and immediately snap-frozen in liquid nitrogen and stored at −80 °C for transcriptome sequencing. An additional six muscle samples from each cage were similarly preserved for lipid extraction and fatty acid composition analysis.

### 2.2. Separation of Sample Lipids

Total lipid extraction of muscle followed a previously reported method, as did diets employed, with slight modifications [20]. Lipids were extracted using a chloroform/methanol (2:1 *v*/*v*) solution, and the chloroform layer was dried under a nitrogen (N_2_) atmosphere to obtain total lipids. To prevent oxidation, 0.02% butylated hydroxytoluene (BHT) was added to solvents. Lipid fractionation was performed as described by He et al. [21]. Briefly, approximately 0.50 g of lipid was dissolved in 20 mL of acetone, followed by multiple extractions (2–3 times) to separate the supernatant and acetone-insoluble fraction. The supernatant was dried under a N_2_ atmosphere in a 50 °C water bath to remove acetone to obtain crude TAG. Ethanol-insoluble fractions were mixed with 95% ethanol (1:10 g/mL), and separated into ethanol-insoluble and supernatant samples. The ethanol-insoluble fraction, presumed to contain PE, was collected and dried under a N_2_ atmosphere to yield a crude PE extract. The supernatant was dried under a N_2_ atmosphere in a 50 °C water bath, mixed with acetone (1:20 g/mL), filtered, and dried under N_2_ to obtain crude PC. TAG, PC, and PE were purified using silica gel GF254 TLC plates (200 mm × 200 mm, Haiyang Chemical Group, Qingdao, China). The developing solvent for TAG was n-hexane/diethyl ether/formic acid (80:20:2, *v*/*v*/*v*), the developing solvent for PE and PC was chloroform/methanol/water (65:25:4, *v*/*v*/*v*). The TAG, PC, and PE were identified by comparison with their respective standards.

### 2.3. Positional Distribution Analysis of Fatty Acid in TAG

Fatty acid composition at the *sn*-2 position of TAG was determined using a modified enzymatic hydrolysis method [22,23]. Briefly, approximately 20 mg of purified TAG was combined with 1 mL of Tris-HCl buffer (1 M, pH 7.6) and 0.25 mL of 0.05% bile salt solution in a 10 mL centrifuge tube. In addition, 0.1 mL of 2.2% CaCl_2_ solution and 10 mg of pancreatic lipase were added to the centrifuge tube and shaken vigorously. The mixture was incubated at 37 °C for 3 min with intermittent shaking (10 s bursts at 2 min intervals). The reaction was terminated by adding 1 mL of 6 M hydrochloric acid, after which 2 mL of diethyl ether was introduced. The sample was centrifuged at 4200 rpm for 5 min, and the upper organic phase was collected and concentrated under a N_2_ atmosphere. The concentrated lipid extract was separated by TLC using n-hexane-diethyl ether-acetic acid (50:50:1, *v*/*v*/*v*) as the developing solvent. The *sn*-2 monoacylglycerol (*sn*-2 MAG) was collected under ultraviolet light, extracted twice with diethyl ether, dried under a N_2_ stream, and subsequently analyzed for fatty acid composition.

### 2.4. Fatty Acid Analysis

Fatty acid analysis of purified TAG, PE, PC, and *sn*-2 MAG was performed as described previously [24]. Briefly, fatty acid methyl esters (FAME) were prepared using a mixture of 0.5 mol L^−1^ KOH in methanol with boron trifluoride etherate. FAME profiles were determined using gas chromatography (GC-7890B, Agilent Technologies, Palo Alto, CA, USA) equipped with a hydrogen flame ionization detector. The GC was equipped with a capillary column (60 m × 0.25 mm CP7487, 0.20 μm film thickness; Agilent Technologies, Santa Clara, CA, USA). By comparing the FAME profiles of the samples with FAME standards (Sigma No. CRM47885, Sigma-Aldrich, St. Louis, MO, USA), the fatty acid species were identified and expressed as percentages of total fatty acids.

Each fatty acids in the *sn*-1/3 positions can be calculated from its concentration in the intact TAG and *sn*-2 MAG, according to the following relationship [23].

*sn*-1/3 positions mol % = (TAG mol % × 3 − *sn*-2 MAG mol %)/2

### 2.5. RNA Extraction, cDNA Library Preparation, and Illumina Sequencing

Transcriptome sequencing was performed by Shanghai Majorbio Bio-pharm Technology Co., Ltd. (Shanghai, China). Total RNA was extracted from the frozen muscle using Trizol reagent (Takara, Shanghai, China). Prior to cDNA library construction, total RNA was first separated by oligo-dT-attached magnetic beads and then fragmented via a fragmentation buffer. Taking these obtained short fragments as templates, double-stranded cDNA was synthesized according to the instructions accompanying a Super Script double-stranded cDNA synthesis kit (Invitrogen, Carlsbad, CA, USA) with random hexamer primers (Illumina, San Diego, CA, USA). Subsequently, the synthesized cDNA was subjected to end-repair, phosphorylation, and “A” base addition in turn, according to Illumina’s library construction protocol. The cDNA target fragments of 200–300 bp were selected on 2% Low Range Ultra Agarose, followed by PCR amplification. Phusion DNA polymerase (New England Biolabs, Boston, MA, USA) was used for PCR amplification (15 cycles). RNA-Seq libraries were sequenced in a single lane on an Illumina Novaseq 6000 sequencer (Illumina, San Diego, CA, USA) after being quantified by TBS380.

### 2.6. De Novo Transcriptome Assembly, Sequence Annotation, and Differentially Expressed Gene Identification

Before de novo transcriptome assembly, quality control of sequencing data was carried out using SeqPrep software (version 1.2, https://github.com/jstjohn/SeqPrep, accessed on 20 October 2020). Raw reads were cleaned by removing reads containing sequencing adapters, reads with over 10% ambiguous “N” nucleotides, and low-quality reads with over 50% of bases with a Q-value of ≤20. The remaining reads were de novo assembled using Trinity software (Version 2.8.5, https://github.com/trinityrnaseq/trinityrnaseq, accessed on 20 October 2020), without a reference genome [25]. After assembly, the results were re-evaluated. Those contigs with chimera, incomplete assembly, and base errors were removed using TransRate package (version 1.0.3, http://hibberdlab.com/transrate, accessed on 20 October 2020) and CD-HIT (http://github.com/weizhongli/cdhit/releases/, accessed on 20 October 2020). The longest transcript from potential alternative splicing transcripts was defined as a unigene.

To annotate the transcriptome, six databases, including the National Center for Biotechnology Information NR database (https://www.ncbi.nlm.nih.gov/, accessed on 20 October 2020), Swiss-Prot protein database (http://web.expasy.org/docs/swiss-prot_guideline.html, accessed on 20 October 2020), Pfam database (http://pfam.xfam.org/, accessed on 20 October 2020), Clusters of Orthologous Groups (COG) of proteins database (http://www.ncbi.nlm.nih.gov/COG/, accessed on 20 October 2020), Gene Ontology (GO) database (http://www.geneontology.org, accessed on 20 October 2020), and Kyoto Encyclopedia of Genes and Genomes (KEGG) database (http://www.genome.jp/kegg/, accessed on 20 October 2020), were searched using BLAST (Version 2.17.0, NCBI, Bethesda, MD, USA), with a cut-off e-value of 10^−5^.

### 2.7. Real-Time PCR

The RNA extraction and cDNA synthesis of muscle were performed following the methods used in our previous studies [24,26]. The expression levels of *gpat1*, *gpat3*, *gpat4*, *agpat1*, *agpat5*, *agpat8*, *dgat1*, *dgat2*, *lpeat1*, *lpgat1*, *lpcat1*, *lpcat2*, *lpcat3,* and *lpcat4* were determined. The primers were designed using Premier 5.0 (Premier Biosoft International, Palo Alto, CA, USA), according to the gene sequences from the genome sequences of *T. ovatus* (Table 1). Real-time PCR was performed via the Real-Time PCR Detection System (CFX96, Bio-Rad Laboratories, Inc., Hercules, CA, USA) [27]. The assays were performed using a total volume of 20 μL, including 10 μL of TB Green™ Premix Ex Taq™ II (TaKaRa, Shanghai, China), containing 0.8 μL of each primer, 1.6 μL of cDNA, and 6.8 μL of DEPC-water. Relative expression levels of the target genes were calculated according to the 2^−ΔΔCt^ method, with *β-actin* as an internal reference.

### 2.8. Statistical Analysis

All statistical analyses were conducted using SPSS software (version 23.0). Prior to analysis, data were tested for normality using the Shapiro–Wilk test and for homogeneity of variances using Levene’s test. Group differences were assessed by one-way ANOVA, followed by Tukey’s post hoc test for multiple comparisons. Results are presented as mean ± standard error of the mean (SEM). Statistical significance was considered at *p* < 0.05. Data analysis was conducted on the free online Majorbio Cloud Platform (www.majorbio.com, accessed on 20 October 2020).

## 3. Results

### 3.1. The Contents of TAG, PC, and PE in Muscle

**Figure 1:**
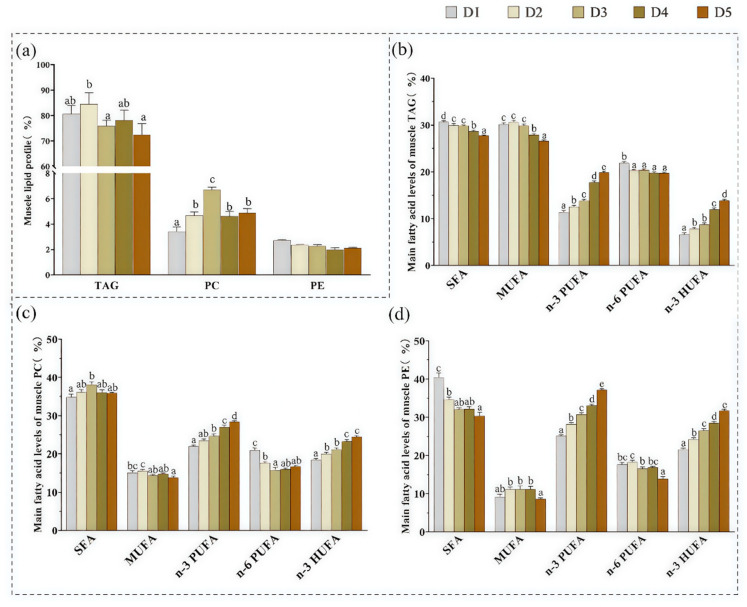
Effects of dietary n-3 HUFA levels on muscle lipid classes and positional distribution of fatty acids in TAG, PC, and PE of *Trachinotus ovatus*. Values represent means of three replicate net cages with two fish per cage (*n* = 3) per treatment. Statistical differences were analyzed using one-way ANOVA, followed by Tukey’s test. Values with different letters are significantly different (*p* < 0.05). (**a**) Contents of TAG, PC, and PE (% total lipid); (**b**–**d**) main fatty acid compositions of TAG, PC, and PE. TAG, triacylglycerol; PC, phosphatidylcholine; PE, phosphatidylethanolamine; SFA, saturated fatty acids; MUFA, monounsaturated fatty acids; PUFA, polyunsaturated fatty acids; HUFA, highly unsaturated fatty acids.

As shown in Figure 1a, TAG content accounted for 72.47% to 84.53% of total lipids, followed by PC (3.41–6.70%), and PE (1.98–2.71%) in all groups. The TAG content in the muscle of groups D3 and D5 was significantly lower than that of group D2 (*p* < 0.05). In contrast, PC content in group D3 was significantly higher than that in the other groups (*p* < 0.05). No significant differences were observed in muscle PE content among the dietary groups.

### 3.2. Fatty Acid Composition of TAG, PC, and PE

As shown in Tables S1–S3, no significant difference (*p* > 0.05) was observed in the 16:1 content of muscle TAG, PC, and PE among all treatments. Values of 16:0, 18:1-n9c, 18:2-n6, and 22:6-n3 were the predominant SFA, MUFA, n-6 PUFA, and n-3 PUFA values in TAG, PC, and PE fatty acids, respectively. By increasing the dietary n-3 HUFA levels from 0.64% (D1) to 2.10% (D5), the contents of 16:0, 18:0, 18:1-n9c, SFA, MUFA, and the n-6/n-3 PUFA ratio in TAG; the 18:2n-6, 18:3n-3, MUFA, n-6 PUFA, and n-6/n-3 PUFA in PC; as well as 16:0, 18:2n-6, SFA, n-6 PUFA, and the n-6/n-3 PUFA ratio in PE were decreased (*p* < 0.05). Conversely, the contents of EPA, DPA, DHA, n-3 PUFA, and n-3 HUFA in TAG, PC, and PE increased with increasing dietary n-3 HUFA levels.

As shown in Figure 1b–d, the contents of SFA, n-3 PUFA, and n-3 HUFA in PC and PE were significantly (*p* < 0.05) higher than those in TAG across all groups, while MUFA and n-6 PUFA contents showed the opposite trend. Regarding the fatty acid compositions of PL, the contents of SFA (except in the D1 and D2 groups) and MUFA in PC were significantly (*p* < 0.05) higher than those in PE across all groups, whereas the opposite was observed for n-3 PUFA and n-3 HUFA.

### 3.3. Fatty Acid Composition and Positional Distribution in Muscle TAG

**Figure 2:**
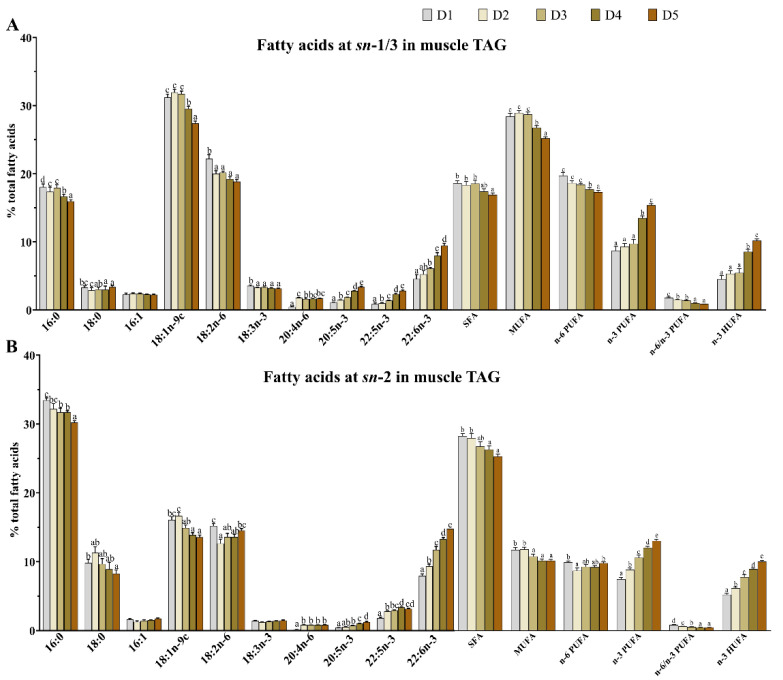
The positional distribution of fatty acids in muscle TAG of *Trachinotus ovatus*. Values represent means of three replicate net cages with two fish per cage (*n* = 3) per treatment. Statistical differences were analyzed using one-way ANOVA, followed by Tukey’s test. Values with different letters are significantly different (*p* < 0.05). (**A**) Fatty acid composition at the *sn*-1/3 position (% of total fatty acids); (**B**) fatty acid composition at the *sn*-2 position (% of total fatty acids). SFA, saturated fatty acid; MUFA, monounsaturated fatty acids; PUFA, polyunsaturated fatty acids; HUFA, highly unsaturated fatty acids.

As shown in Figure 2, no significant difference (*p* > 0.05) was observed in the contents of 18:0 and 16:1 in the *sn*-1/3 positions of TAG among all treatments. As the n-3 HUFA levels increased from 0.64% (D1) to 2.10% (D5), the contents of 16:0, 18:1, 18:3n-3, SFA, MUFA, n-6 PUFA, and the n-6/n-3 PUFA ratio in the *sn*-1/3 positions of TAG, as well as the 16:0, 18:0, 18:1, SFA, MUFA, and the n-6/n-3 PUFA ratio in the *sn*-2 position of TAG, all decreased significantly (*p* < 0.05). Conversely, the contents of EPA, DPA, DHA, n-3 PUFA, and n-3 HUFA in both the *sn*-1/3 and *sn*-2 positions of muscle TAG increased significantly (*p* < 0.05).

**Figure 3:**
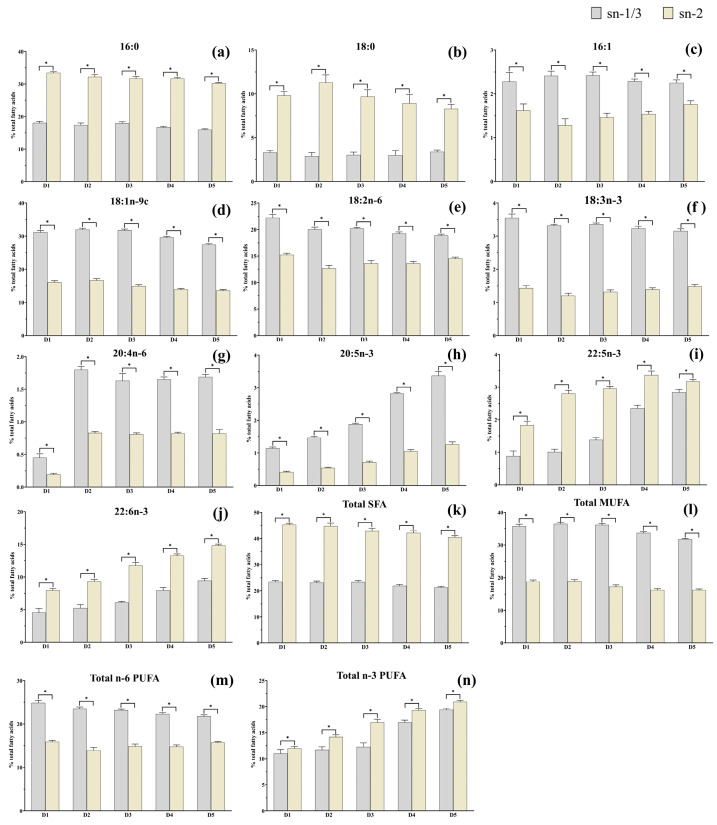
The positional distribution of fatty acids in muscle TAG of *Trachinotus ovatus*. Values represent means of three replicate net cages with two fish per cage (*n* = 3) per treatment. (**a**) 16:0; (**b**) 18:0; (**c**) 16:1; (**d**) 18:1n-9c; (**e**) 18:2n-6; (**f**) 18:3n-3; (**g**) 20:4n-6; (**h**) 20:5n-3; (**i**) 22:5n-3; (**j**) 22:6n-3; (**k**) Total SFA; (**l**) Total MUFA; (**m**) Total n-6 PUFA; (**n**) Total n-3 PUFA. Statistical differences were analyzed using one-way ANOVA, followed by Tukey’s test. Values with “*” show significant differences (*p* < 0.05) between the outer position (*sn*-1/3) and the inner position (*sn*-2). SFA, saturated fatty acid; MUFA, monounsaturated fatty acids; PUFA, polyunsaturated fatty acids.

As shown in Figure 3, the contents of 16:0, 18:0, DPA, DHA, SFA, and n-3 PUFA in the *sn*-2 position of TAG were significantly (*p* < 0.05) higher than those in the *sn*-1/3 positions in all groups. In contrast, the contents of 16:1, 18:1n-9c, 18:2n-6, 18:3n-3, 20:4n-6, EPA, MUFA, and n-6 PUFA were significantly (*p* < 0.05) higher in the *sn*-1/3 positions compared to in the *sn*-2 position.

### 3.4. Transcriptome Sequencing, Assembly, and Annotation

As can be seen from Table S6, 43,631,590 and 45,273,460 clean reads were obtained from the D2 and D5 group, respectively. After de novo assembly, 102,235,850 bp with 126,792 unigenes were obtained in the D2 group, with a GC content of 46.52%. The clean reads were matched with their corresponding assembly sequences, and the mean mapped reads was 1844.

As shown in Table S7, 126,792 assembled unigenes were annotated via the BLAST tool using six public databases. The 51,624 (40.72%), 60,585 (47.78%), 50,516 (39.84%), 17,807 (14.04%), 17,928 (14.14%), and 47,171 (37.20%) unigenes were matched using NR, Swiss-Prot, Pfam, COG, GO, and KEGG, respectively.

### 3.5. Function Enrichment Analysis of GO Terms and KEGG Pathway

**Figure 4:**
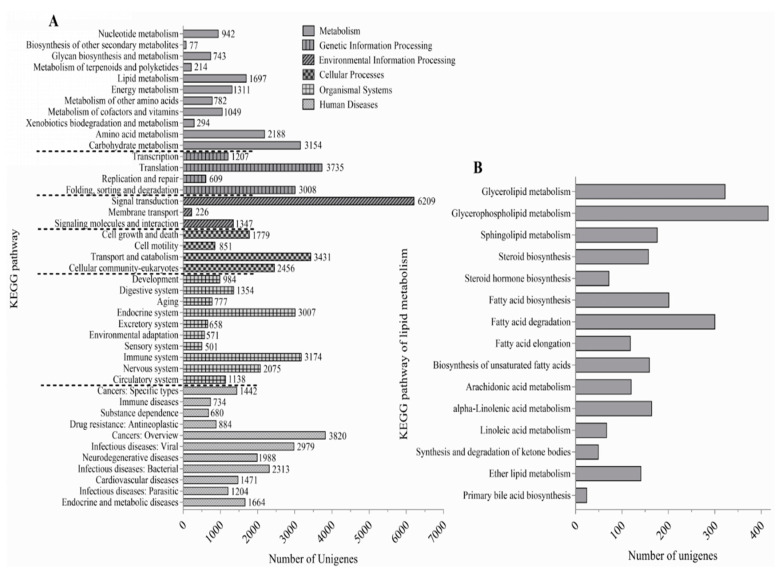
The total unigenes (**A**) and lipid metabolism-related unigenes (**B**) of *Trachinotus ovatus* muscle were enriched by the KEGG pathway.

As seen in the Figure 4, 126,792 unigenes were primarily associated with metabolism, genetic information processing, environmental information processing, cellular processes, organismal systems, and human diseases (Figure 4A). As for metabolism pathways, the number of unigenes in carbohydrate metabolism (3154) was the highest, followed by that in amino acid metabolism (2188) and lipid metabolism (1697). Notably, the lipid metabolism-related unigenes were enriched in 15 pathways relating to glycerolipid metabolism, glycerophospholipid metabolism, and sphingolipid metabolism (Figure 4B).

### 3.6. Differentially Expressed Unigenes and Functional Enrichment

**Figure 5:**
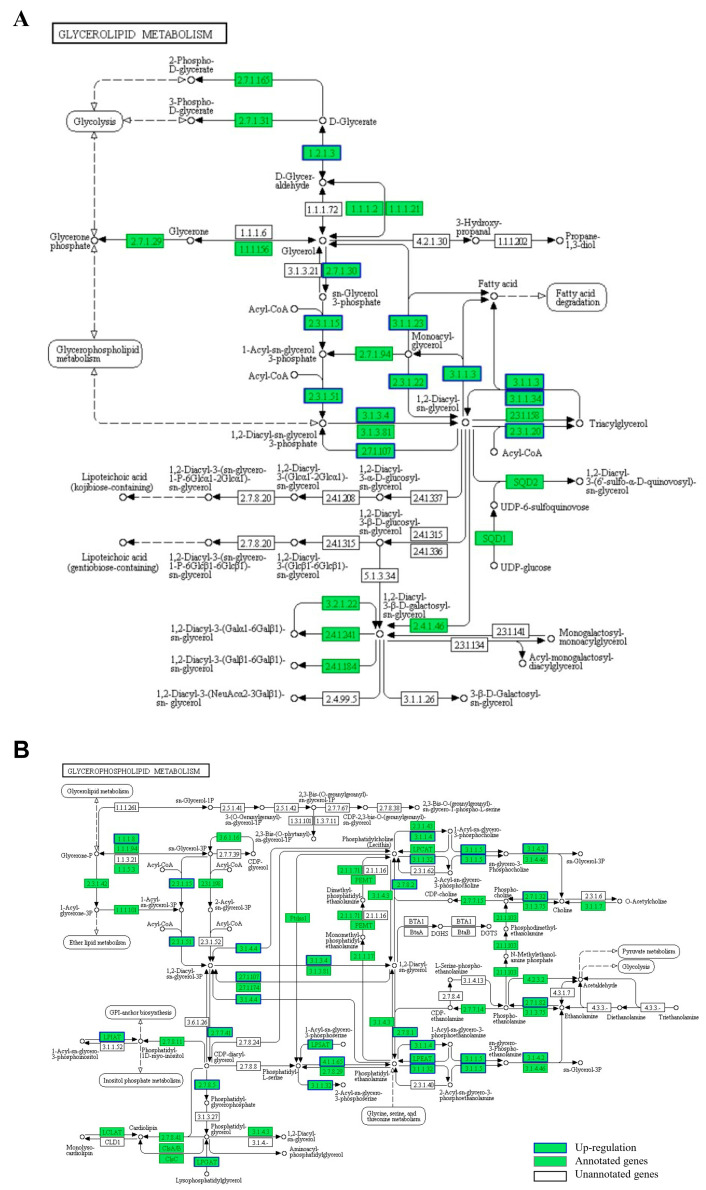
Enrichment of differentially expressed genes involved in triglyceride (**A**) and phospholipid (**B**) metabolic pathways. Reaction processes (solid arrows); metabolic pathways (dashed arrows).

As can be seen from Figure 5, KEGG pathway enrichment analysis of the upregulated differentially expressed genes revealed significant involvement in triglyceride and phospholipid metabolic pathways. In the de novo synthesis pathways, we observed upregulation of all key positional acyltransferases, i.e., *gpat* (*sn*-1), *agpat* (*sn*-2), and *dgat* (*sn*-3), which sequentially esterify fatty acids to the glycerol backbone. Additionally, glycerophospholipid remodeling enzymes including *lpcat*, *lpeat*, and *lpgat* were similarly upregulated.

### 3.7. Expression of Genes Related to Fatty Acid Deposition and Positional Distribution

**Figure 6:**
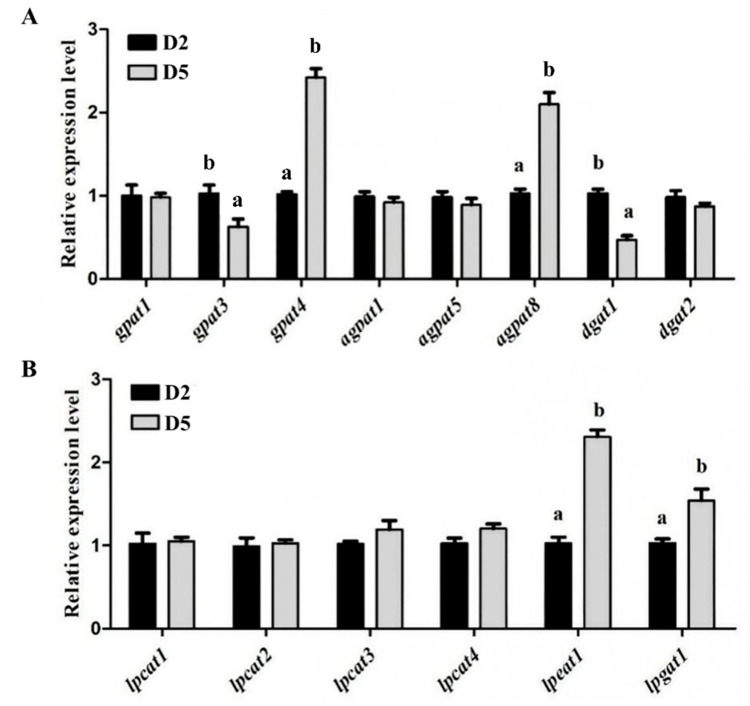
Validation of differentially expressed genes by qPCR. Values represent means of three replicate net cages with two fish per cage (*n* = 3) per treatment. Statistical differences were analyzed using one-way ANOVA, followed by Tukey’s test. Columns marked by different letters indicate significant differences (*p* < 0.05). (**A**) *gapt1*, *gapt2*, *gapt3*, *agpat1*, *agpat5*, *agpat8*, *dgat1, dgat2*. (**B**) *lpcat1*, *lpcat2*, *lpcat3, lpcat4*, *lpeat1*, *lpgat1*. *gpat*, Glycerol-3-phosphate acyltransferase; *agpat*, 1-acyl-*sn*-glycerol-3-phosphate acyltransferase; *dgat*, Diacylglycerol acyltransferase; *lpeat*, Lysophosphatidylethanolamine acyltransferase; *lpgat*, Lysophosphatidylglycerol acyltransferase; *lpcat*, Lysophosphatidylcholine acyltransferase.

As can be seen from Figure 6, no statistical difference (*p* > 0.05) was observed in the expression levels of *gapt1*, *agpat1*, *agpat5*, *dgat2*, *lpcat1*, *lpcat2*, *lpcat3,* and *lpcat4* among the two treatments. However, the expression levels of *gpat4*, *agpat8*, *lpeat1,* and *lpgat1* in the muscle of the D5 group were all significantly (*p* < 0.05) higher than those of the D2 group, whereas the opposite was found for *gpat3* and *dgat1* expression levels.

## 4. Discussion

In this study, TAG was identified as the predominant lipid class in muscle, ranging from 72.47% to 84.53%, followed by PC (3.41–6.70%) and PE (1.98–2.71%), across all groups. Increasing dietary levels of n-3 HUFA significantly elevate the contents of EPA and DHA in muscle PC and PE molecules. Notably, the n-3 HUFA concentrations in PL, comprising PC and PE, are markedly higher than those in TAG, with PE exhibiting significantly greater contents than PC, indicate that n-3 HUFA is preferentially esterified on PL, independent of dietary contribution. This fact reflects the higher affinity of acylases and transacylases esterifying fatty acids into the different phosphoacylglycerides for n-3 HUFA. The preference for PL incorporation may be due to their role in enhancing the stability of the PL bilayer in cell membranes, increasing oxidative fiber levels, and facilitating the transition from fast to slow fiber types [28]. This may be attributed to the ability of n-3 HUFA to increase the number of fat cells without increasing their volume, thereby improving PL content. This finding is further supported by the high muscle PL content observed in *T. ovatus* fed with diet D3. In addition, similar results were also observed in *Oreochromis niloticus* [5] and *Gadus morhua* [29], where fish fed diets with higher HUFA levels exhibited increased PL content. This result was consistent with the report for *Bostrichthys sinensis*, *Erythroculter ilishaeformis*, *Siniperca kneri Garman*, *Squaliobarbus curriculus*, *Pseudobagrus fulvidraco* [23], and *S. salar* [11]. Therefore, the predominant lipid molecule, TAG, PC, and PE were analyzed in greater detail.

The positional distribution of fatty acids within lipid molecules plays a critical role in determining the nutritional value and physiological metabolic functions of lipids [26]. In the present study, the contents of EPA, DPA, DHA, n-3 PUFA, and n-3 HUFA in lipid molecules, as well as in the *sn*-1/3 and *sn*-2 positions of muscle TAG, increased as dietary n-3 HUFA levels rose from 0.64% to 2.10%. These results are consistent with those reported in *O. niloticus* [5] and *S. salar* [6,30] that were fed diets including vegetable oil (VO) and FO. Meanwhile, the content of TAG containing 16:0 at the *sn*-2 position in muscle was significantly higher than those of the *sn*-1/3 positions across all groups, indicating that 16:0 was preferentially distributed at the *sn*-2 position of the glycerol backbone in TAG (irrespective of dietary fatty acid composition). This result was consistent with that reported in *T. ovatus* [21] and mammals (from lard and bovine and human milk) [31,32]. Moreover, 16:0 at the *sn*-2 position of TAG has been shown to enhance nutrient absorption in infants [33] and is less likely to form insoluble soaps with magnesium and calcium in the human body [34]. Meanwhile, the contents of DPA and DHA in the TAG *sn*-2 position were significantly higher than those in the *sn*-1/3 positions across all groups, consistent with findings in *Ictalurus punctatus*, *Ictalurus melas*, and *Micropterus salmoides* [35]. This suggests that the positional distribution of DPA and DHA are preferably esterified at the *sn*-2 positions of TAG. However, the preferential distribution of DHA at the *sn*-2 position of TAG has been reported to enhance lipid absorption and physiological responses [8]. These results indicate that *T. ovatus* fillets exhibit extremely high nutritional value due to their relatively high contents of 16:0, DPA, and DHA at *sn*-2 position of TAG.

The positional distribution of 16:1, 18:1n-9c, 18:2n-6, 18:3n-3, 20:4n-6, and EPA was predominantly esterified at the *sn*-1/3 positions in the TAG of *T. ovatus* fillets across all the groups. Previous studies have suggested that 18:1n-9c may contribute to the prevention of breast cancer [36] and colorectal cancer [37], and it has been inversely associated with both systolic and diastolic blood pressure [38]. However, the potential physiological effects of its positional distribution in TAG remain unclear and warrant further investigation. Both 18:3n-3 and 20:4n-6 have been shown to play anti-inflammatory roles in humans [39]. The present study demonstrated that their specific deposition at the *sn*-1/3-position of TAG was more readily released than that at the *sn*-2 position in *T. ovatus* muscle, which indicated that these fillets are a good source of 18:3n-3 and 20:4n-6 for human consumption. In our study, unsaturated fatty acids (UFA), including MUFA and n-6 PUFA, were preferentially esterified at the *sn*-1/3 positions, while SFA was mainly distributed at the *sn*-2 position of TAG across all dietary groups. This positional distribution pattern is consistent with previous findings in *T. ovatus* [21], further highlighting the high nutritional value of its muscle tissue. Notably, this is supported by the fact that infants’ absorption of breast milk lipids, which play a critical role in infant growth and development, is better due to its *sn*-USU (U stands for UFA; S stands for SFA)-containing TAG composition [40,41]. These findings suggest that *T. ovatus* fillets could serve as a potential complementary dietary source of essential fatty acids for infants.

To explore the molecular mechanisms underlying HUFA deposition in *T. ovatus*, RNA-Seq analysis revealed the enrichment of 1697 unigenes in 15 lipid metabolism-related pathways, including glycerolipid metabolism, glycerophospholipid metabolism, and sphingolipid metabolism. These pathways are closely associated with HUFA incorporation into tissue lipids. Notably, previous studies have shown that enhanced glycerolipid and glycerophospholipid synthesis contributes to increased tissue HUFA levels [21,41]. Comparative analysis between fish fed the D2 and D5 diets identified 232 differentially expressed genes (DEGs), including 119 upregulated and 113 downregulated genes. KEGG pathway enrichment of these DEGs further highlighted glycerophospholipid metabolism as the most significantly involved process, followed by glycerolipid and sphingolipid metabolism. These findings suggest that these lipid metabolic pathways play crucial roles in regulating the lipid composition and TAG structure in *T. ovatus* under varying dietary n-3 HUFA conditions.

The RNA-Seq results of this study demonstrated that elevated dietary n-3 HUFA levels upregulated the expression of *gpat*, *agpat*, *dgat*, *lpsat*, *lpeat,* and *lpgat* during lipid synthesis in the muscle of *T. ovatus*. qPCR validation of these genes revealed significantly higher expression levels of *gpat4*, *agpat8*, *lpeat1,* and *lpgat1* in the muscle of the high n-3 HUFA group compared to the low n-3 HUFA group, consistent with the transcriptomic profiling. These findings suggest that these genes may play a role in regulating HUFA deposition, potentially contributing to lipid remodeling and muscle quality improvement in *T. ovatus*. This may be supported by the following facts: (1) *gpat4*, the rate-limiting enzyme for TAG synthesis in the endoplasmic reticulum, exhibits bifunctional activity by not only catalyzing the initial acylation step in glycerolipid synthesis but also by producing *sn*-2 MAGs through its phosphatase activity [42,43]. Research using GPAT4-KO mice revealed a striking shift in lipid composition, characterized by increased PUFA and decreased MUFA in TAG, DAG, and PL, suggesting that *gpat4* displays a substrate preference for MUFA over PUFA during glycerolipid synthesis [44]. However, its overexpression ultimately leads to the depletion of MUFA precursors and activation of the HUFA biosynthesis pathway. (2) *agpat8* participated in the formation of 1,2-diacyl-*sn*-glycerol-3-phosphate (namely, phosphatidate), which is a precursor for PL [45]; Imae et al. [46] reported that *agpat8* mainly introduced C18:0 into the *sn*-1 position of *sn*-2-acyl LPI, and AGPAT8-KO mouse tissues showed decreased *agpat8* activities toward *sn*-2-acyl LPI and displayed decreased amounts of C18:0-containing PI. These results indicate that *agpat8* is an *sn*-1-position-remodeling enzyme. (3) *lpeat1* and *lpgat1* actively participate in the phospholipid remodeling pathways [47].

The upregulation of *gpat4*, *agpat8*, *lpeat1*, and *lpgat1* may facilitate HUFA incorporation at both the *sn*-1 and *sn*-1/3 positions of TAG through potentially complementary mechanisms. *gpat4* and *agpat8* drive de novo HUFA incorporation during initial glycerolipid synthesis, while *lpeat1* and *lpgat1* promote HUFA enrichment via phospholipid remodeling. This coordinated activity establishes multiple entry points for HUFA integration, resulting in their simultaneous accumulation at terminal TAG positions, while preserving the characteristic *sn*-2 HUFA preference. Although no significant differences in *agpat3* expression levels were observed among treatment groups in the muscle of *T. ovatus* in the present study, mammalian studies have demonstrated that *agpat3* specifically binds to HUFA to generate HUFA-enriched phosphatidic acid [48,49]. Notably, previous research in fish has reported that dietary supplementation with high HUFA levels significantly upregulates the expression of both the *pparγ* and *agpat3* genes in muscle tissue, concomitant with a marked increase in muscle HUFA content [26]. These findings collectively suggest that *agpat3* may play a regulatory role in HUFA deposition in fish species.

## 5. Conclusions

In conclusion, this study demonstrated that dietary n-3 HUFA levels influence the fatty acid composition of TAG, PC, and PE in the muscle of *T. ovatus*. The n-3 HUFA contents in TAG, PC, and PE were positively correlated with dietary intake and showed preferential deposition in PL and at the *sn*-2 position of TAG. Additionally, RNA-Seq analysis identified 126,792 unigenes in *T. ovatus* and highlighted glycerophospholipid and glycerolipid metabolism as key pathways potentially involved in regulating lipid structural characteristics. The genes *gpat4*, *agpat8*, *lpeat1*, and *lpgat1*, which are involved in the esterification of fatty acids to the glycerol backbone, were identified as potential candidates regulating HUFA structural remodeling.

These findings offer new insights into the molecular basis of lipid structural regulation in marine fish muscle and provide a scientific basis for improving the nutritional quality of aquaculture products. Future research should focus on the functional validation of these candidate genes, further explore the specific mechanisms by which diet regulates lipid metabolism and structure, and assess these regulatory effects across different species, developmental stages, and environmental conditions. Such efforts will contribute to a deeper understanding of lipid metabolism and support the development of more effective nutritional strategies in sustainable aquaculture.

## Figures and Tables

**Table 1:** Primer sequences for real-time PCR amplification of muscle genes.

Genes	Primers	Primer Sequences	Annealing Temperature (°C)	Annealing Time (s)	Accession No./Reference
*gpat1*	*gpat1*-F	GAGTCCGACTACACCCAGAG	60	30	Genome sequences
	*gpat1*-R	GTGGTCACCTTGCTCTCTCT			
*gpat3*	*gpat3*-F	GTGGTGTTTGGCTGTAGGTG	60	30	Genome sequences
	*gpat3*-R	GCTCCATCTCCTCCTCCATC			
*gpat4*	*gpat4*-F	CATTGCCAAAGAGCCAACCT	60	30	Genome sequences
	*gpat4*-R	GGTCAGCAGATTCCAGGACT			
*agpat1*	*agpat1*-F	TAGGAGAAAGAGAGGCGGGA	60	30	Genome sequences
	*agpat1*-R	TCGAGACAAAGGGGATGACG			
*agpat5*	*agpat5*-F	AGCACCGATGTACCTTGTCA	60	30	Genome sequences
	*agpat5*-R	TGGCCCTTCATGGTCTCAAT			
*agpat8*	*agpat8*-F	CAGCCTGCCGAAATTTGAGT	60	30	Genome sequences
	*agpat8*-R	CAAGATGAGGTGGCGTTCTG			
*dgat1*	*dgat1*-F	TCCTCAACTGGTGTGTGGTT	60	30	Genome sequences
	*dgat1*-R	TGGTACCCACAGCTAAACGT			
*dgat2*	*dgat2*-F	TAAAACCCACAACCTGCTGC	60	30	Genome sequences
	*dgat2*-R	CGGGACAGATACCTCCAGAC			
*lpeat1*	*lpeat1*-F	CTGTGTGTTATGACCGAGCC	60	30	Genome sequences
	*lpeat1*-R	GTAGTGGCGAACGGATTTCC			
*lpgat1*	*lpgat1*-F	AGTGGATAGTGCTGTTCCCC	60	30	Genome sequences
	*lpgat1*-R	TTACCCACTGCAGGCCTTTA			
*lpcat1*	*lpcat1*-F	GCTTGGATGACTTCGCTCAG	60	30	Genome sequences
	*lpcat1*-R	CCATCCTCCTCTGCCTCAAA			
*lpcat2*	*lpcat2*-F	TTTGCCAGCAGAGTGAGAGA	60	30	Genome sequences
	*lpcat2*-R	CGTTGTCCCACTTCAGTTGG			
*lpcat3*	*lpcat3*-F	CGTTCTGGTATCGCTGTGTG	60	30	Genome sequences
	*lpcat3*-R	GAGCCACACCTTCATGTTGG			
*lpcat4*	*lpcat4*-F	GGCTGGTATTCCCTCCACTT	60	30	Genome sequences
	*lpcat4*-R	CCCGCTTTGTCAAACCATCA			
*β-actin*	*β-actin*-F	TACGAGCTGCCTGACGGACA	60	30	KX987228
	*β-actin*-R	GGCTGTGATCTCCTTCTGC			

*gpat*, Glycerol-3-phosphate acyltransferase; *agpat*, 1-acyl-*sn*-glycerol-3-phosphate acyltransferase; *dgat*, Diacylglycerol acyltransferase; *lpeat*, Lysophosphatidylethanolamine acyltransferase, *lpgat*, Lysophosphatidylglycerol acyltransferase; *lpcat*, Lysophosphatidylcholine acyltransferase.

## Data Availability

The original contributions presented in this study are included in the article/Supplementary Materials. Further inquiries can be directed to the corresponding authors.

## Supplementary Materials

The following supporting information can be downloaded at: https://www.mdpi.com/article/10.3390/ani15162427/s1, Table S1: Fatty acid profiles of muscle triacylglycerols (TAG) of golden pompano *T. ovatus* fed different n-3 HUFA levels (% total fatty acids); Table S2: Fatty acid profiles of muscle phosphatidylcholine (PC) of *T. ovatus* fed different n-3 HUFA levels (% total fatty acids); Table S3: Fatty acid profiles of muscle phosphatidylethanolamine (PE) of *T. ovatus* fed different n-3 HUFA levels (% total fatty acids); Table S4: The *sn*-1/3 position of fatty acids in muscle triacylglycerols (TAG) of *T. ovatus* fed different n-3 HUFA levels (% total fatty acids); Table S5: The *sn*-2 position of fatty acids in muscle triacylglycerols (TAG) of *T. ovatus* fed different n-3 HUFA levels (% total fatty acids); Table S6: Clean data, quality control and summary of Illumina Paired-end sequencing and assembly; Table S7: The result of annotation on unigenes in different databases for *T. ovatus.*
