# Supplementary material for: Transcriptomic Analysis Reveals Regulatory Responses of Fatty Acid Positional Distribution in Triacylglycerols and Lipid Composition to Dietary n-3 HUFA in the Muscle of Trachinotus ovatus
Source: Animals (Basel). 2025 Aug 19;15(16):2427. doi: 10.3390/ani15162427 (PMC12383132; doi:10.3390/ani15162427)
Supplement: Supplementary file 1 [file animals-15-02427-s001.zip › animals-3773192-supplementary.pdf]

Table S1. Fatty acid profiles of muscle triacylglycerols (TAG) of golden *Trachinotus ovatus* fed different n-3 HUFA levels (% total fatty acids).

Item	Groups					*P* value
	D1	D2	D3	D4	D5	
16:0	23.19 ± 0.34^{d}	22.31 ± 0.25^{c}	22.53 ± 0.10^{c}	21.68 ± 0.17^{b}	20.73 ± 0.10^{a}	<0.01
18:0	5.47 ± 0.15^{bc}	5.70 ± 0.07^{c}	5.25 ± 0.12^{ab}	4.97 ± 0.06^{a}	5.01 ± 0.06^{a}	<0.01
16:1	2.06 ± 0.12	2.03 ± 0.03	2.10 ± 0.07	2.04 ± 0.02	2.08 ± 0.06	<0.01
18:1n-9c	26.17 ± 0.39^{c}	26.86 ± 0.41^{c}	26.14 ± 0.30^{c}	24.34 ± 0.23^{b}	22.79 ± 0.29^{a}	<0.01
18:2n-6	19.89 ± 0.37^{b}	17.57 ± 0.12^{a}	17.99 ± 0.12^{a}	17.36 ± 0.15^{a}	17.42 ± 0.13^{a}	<0.01
20:4n-6	0.36 ± 0.03^{a}	1.48 ± 0.04^{c}	1.30 ± 0.07^{b}	1.37 ± 0.03^{bc}	1.40 ± 0.04^{bc}	<0.01
18:3n-3	2.84 ± 0.08^{b}	2.62 ± 0.03^{a}	2.68 ± 0.04^{a}	2.62 ± 0.05^{a}	2.60 ± 0.03^{a}	<0.01
20:5n-3 (EPA)	0.90 ± 0.03^{a}	1.16 ± 0.01^{b}	1.49 ± 0.01^{c}	2.23 ± 0.02^{d}	2.67 ± 0.11^{e}	<0.01
22:5n-3 (DPA)	1.19 ± 0.09^{a}	1.60 ± 0.03^{b}	1.90 ± 0.03^{c}	2.69 ± 0.07^{d}	2.95 ± 0.06^{e}	<0.01
22:6n-3 (DHA)	5.69 ± 0.38^{a}	6.62 ± 0.32^{ab}	7.25 ± 0.38^{b}	9.73 ± 0.30^{c}	11.24 ± 0.22^{d}	<0.01
SFA	30.71 ± 0.21^{d}	29.83 ± 0.50^{c}	29.87 ± 0.08^{c}	28.66 ± 0.17^{b}	27.71 ± 0.08^{a}	<0.01
MUFA	30.12 ± 0.42^{c}	30.61 ± 0.41^{c}	29.86 ± 0.38^{c}	27.90 ± 0.25^{b}	26.59 ± 0.25^{a}	<0.01
n-6 PUFA	21.88 ± 0.34^{b}	20.31 ± 0.13^{a}	20.42 ± 0.07^{a}	19.80 ± 0.14^{a}	19.77 ± 0.13^{a}	<0.01
n-3 PUFA	11.32 ± 0.45^{a}	12.53 ± 0.32^{b}	13.84 ± 0.36^{c}	17.76 ± 0.30^{d}	19.90 ± 0.18^{e}	<0.01
n-6/n-3 PUFA	1.95 ± 0.10^{c}	1.62 ± 0.03^{b}	1.48 ± 0.04^{b}	1.12 ± 0.02^{a}	0.99 ± 0.01^{a}	<0.01
n-3 HUFA	6.59 ± 0.40^{a}	7.78 ± 0.32^{b}	8.74 ± 0.38^{b}	11.96 ± 0.29^{c}	13.91 ± 0.23^{d}	<0.01

Values represent means of 3 replicate net cages with 2 fish per cage (n = 3) per treatment. Values in each row without sharing a common letter are significantly different ($P < 0.05$). SFA, saturated fatty acid; MUFA, monounsaturated fatty acid; PUFA, polyunsaturated fatty acid; HUFA, high unsaturated fatty acids.

Table S2. Fatty acid profiles of muscle phosphatidylcholine (PC) of *Trachinotus ovatus* fed different n-3 HUFA levels (% total fatty acids).

Item	Groups					*P* value
	D1	D2	D3	D4	D5	
16:0	18.83 ± 0.87	18.63 ± 0.36	19.70 ± 0.76	19.57 ± 0.10	19.64 ± 0.38	0.03
18:0	15.26 ± 0.63^{a}	16.99 ± 0.40^{bc}	17.73 ± 0.24^{c}	16.06 ± 0.63^{ab}	15.75 ± 0.29^{ab}	<0.01
16:1	0.40 ± 0.02	0.35 ± 0.03	0.42 ± 0.04	0.41 ± 0.03	0.42 ± 0.02	<0.01
18:1n-9c	12.96 ± 0.49^{bc}	13.55 ± 0.28^{c}	10.91 ± 1.01^{a}	12.73 ± 0.16^{bc}	11.67 ± 0.31^{ab}	<0.01
18:2n-6	16.82 ± 0.69^{d}	11.10 ± 0.14^{c}	9.46 ± 0.64^{b}	9.59 ± 0.14^{b}	10.07 ± 0.23^{b}	<0.01
20:4n-6	1.79 ± 0.07^{a}	5.06 ± 0.10^{b}	5.06 ± 0.14^{b}	5.09 ± 0.23^{b}	5.33 ± 0.11^{b}	<0.01
18:3n-3	1.33 ± 0.05^{c}	1.15 ± 0.04^{b}	0.95 ± 0.05^{a}	1.07 ± 0.02^{ab}	1.00 ± 0.01^{ab}	<0.01
20:5n-3 (EPA)	1.75 ± 0.04^{a}	1.74 ± 0.05^{a}	1.90 ± 0.11^{a}	2.25 ± 0.07^{b}	2.26 ± 0.07^{b}	<0.01
22:5n-3 (DPA)	1.84 ± 0.05^{a}	1.97 ± 0.10^{a}	2.23 ± 0.10^{b}	2.38 ± 0.08^{bc}	2.56 ± 0.05^{c}	<0.01
22:6n-3 (DHA)	16.70 ± 0.26^{a}	18.26 ± 0.38^{b}	19.20 ± 0.49^{b}	20.97 ± 0.44^{c}	22.22 ± 0.25^{d}	<0.01
SFA	34.78 ± 0.87^{a}	36.07 ± 0.77^{ab}	38.07 ± 0.79^{b}	36.04 ± 0.71^{ab}	35.87 ± 0.19^{ab}	0.02
MUFA	15.04 ± 0.55^{bc}	15.55 ± 0.29^{c}	14.39 ± 0.09^{ab}	14.75 ± 0.19^{ab}	13.79 ± 0.29^{a}	0.01
n-6 PUFA	20.90 ± 0.73^{c}	17.63 ± 0.24^{b}	15.68 ± 0.86^{a}	16.01 ± 0.28^{ab}	16.71 ± 0.24^{ab}	<0.01
n-3 PUFA	21.96 ± 0.28^{a}	23.41 ± 0.49^{ab}	24.64 ± 0.59^{b}	26.96 ± 0.55^{c}	28.40 ± 0.26^{d}	<0.01
n-6/n-3 PUFA	0.95 ± 0.03^{c}	0.75 ± 0.01^{b}	0.64 ± 0.04^{a}	0.59 ± 0.00^{a}	0.59 ± 0.01^{a}	<0.01
n-3 HUFA	18.45 ± 0.26^{a}	20.00 ± 0.39^{b}	21.10 ± 0.50^{b}	23.22 ± 0.49^{c}	24.47 ± 0.28^{c}	<0.01

Values represent means of 3 replicate net cages with 2 fish per cage (n = 3) per treatment. Values in each row without sharing a common letter are significantly different ($P < 0.05$). SFA, saturated fatty acid; MUFA, monounsaturated fatty acid; PUFA, polyunsaturated fatty acid; HUFA, high unsaturated fatty acids.

Table S3. Fatty acid profiles of muscle phosphatidylethanolamine (PE) of *Trachinotus ovatus* fed different n-3 HUFA levels (% total fatty acids).

Item	Groups					*P* value
	D1	D2	D3	D4	D5	
16:0	24.32 ± 0.57[c]	21.80 ± 0.44[b]	20.86 ± 0.13[b]	20.26 ± 0.35[b]	17.72 ± 0.61[a]	<0.01
18:0	16.05 ± 1.08[b]	12.76 ± 0.40[a]	11.21 ± 0.41[a]	11.92 ± 0.32[a]	12.61 ± 0.44[a]	<0.01
16:1	0.25 ± 0.03	0.29 ± 0.03	0.26 ± 0.04	0.31 ± 0.01	0.35 ± 0.05	<0.01
18:1n-9c	7.54 ± 0.78[ab]	9.03 ± 0.67[b]	8.96 ± 0.95[b]	9.07 ± 0.74[b]	6.56 ± 0.25[a]	<0.01
18:2n-6	13.41 ± 0.45[d]	11.92 ± 0.60[c]	9.91 ± 0.28[b]	10.19 ± 0.21[b]	7.97 ± 0.44[a]	<0.01
20:4n-6	2.11 ± 0.12[a]	4.49 ± 0.12[b]	4.65 ± 0.14[b]	4.61 ± 0.11[b]	4.53 ± 0.17[b]	<0.01
18:3n-3	1.23 ± 0.10	1.08 ± 0.05	0.96 ± 0.13	1.21 ± 0.14	1.37 ± 0.22	0.36
20:5n-3 (EPA)	1.50 ± 0.06[a]	1.97 ± 0.15[b]	2.38 ± 0.14[c]	2.86 ± 0.11[d]	3.45 ± 0.11[e]	<0.01
22:5n-3 (DPA)	1.78 ± 0.06[a]	2.37 ± 0.14[b]	2.63 ± 0.04[bc]	2.83 ± 0.12[cd]	3.25 ± 0.11[e]	<0.01
22:6n-3 (DHA)	20.07 ± 0.32[a]	22.26 ± 0.32[b]	24.11 ± 0.57[c]	25.64 ± 0.27[d]	28.32 ± 0.34[e]	<0.01
SFA	40.38 ± 1.20[c]	34.56 ± 0.75[b]	32.08 ± 0.43[ab]	32.18 ± 0.56[ab]	30.33 ± 0.99[a]	<0.01
MUFA	9.08 ± 0.85[ab]	11.04 ± 0.69[b]	11.11 ± 0.92[b]	11.14 ± 0.75[b]	8.60 ± 0.24[a]	0.04
n-6 PUFA	17.62 ± 0.54[bc]	18.19 ± 0.60[c]	16.53 ± 0.41[b]	16.88 ± 0.19[bc]	13.85 ± 0.65[a]	<0.01
n-3 PUFA	25.09 ± 0.16[a]	28.17 ± 0.33[b]	30.72 ± 0.53[c]	33.03 ± 0.34[d]	37.19 ± 0.21[e]	<0.01
n-6/n-3 PUFA	0.70 ± 0.02[d]	0.65 ± 0.02[c]	0.54 ± 0.01[b]	0.51 ± 0.01[b]	0.37 ± 0.02[a]	<0.01
n-3 HUFA	21.57 ± 0.29[a]	24.23 ± 0.37[b]	26.49 ± 0.54[c]	28.50 ± 0.32[d]	31.77 ± 0.39[e]	<0.01

Values represent means of 3 replicate net cages with 2 fish per cage (n = 3) per treatment. Values in each row without sharing a common letter are significantly different ($P < 0.05$). SFA, saturated fatty acid; MUFA, monounsaturated fatty acid; PUFA, polyunsaturated fatty acid; HUFA, high unsaturated fatty acids.

Table S4. The *sn*-1/3 position of fatty acids in muscle triacylglycerols (TAG) of *Trachinotus ovatus* fed different n-3 HUFA levels (% total fatty acids).

Item	Groups					*P* value
	D1	D2	D3	D4	D5	
16:0	18.06 ± 0.46^{d}	17.38 ± 0.66^{cd}	17.95 ± 0.45^{bc}	16.68 ± 0.30^{ab}	15.97 ± 0.22^{a}	<0.01
18:0	3.30 ± 0.24	2.89 ± 0.41	3.03 ± 0.32	2.99 ± 0.53	3.38 ± 0.21	0.95
16:1	2.28 ± 0.21	2.41 ± 0.11	2.42 ± 0.08	2.29 ± 0.05	2.25 ± 0.07	<0.01
18:1n-9c	31.20 ± 0.50^{c}	31.96 ± 0.46^{c}	31.75 ± 0.39^{c}	29.57 ± 0.35^{b}	27.41 ± 0.33^{a}	<0.01
18:2n-6	22.22 ± 0.61^{b}	20.02 ± 0.43^{a}	20.19 ± 0.21^{a}	19.25 ± 0.31^{a}	18.86 ± 0.29^{a}	<0.01
20:4n-6	0.45 ± 0.06^{a}	1.80 ± 0.05^{b}	1.63 ± 0.11^{ab}	1.65 ± 0.04^{ab}	1.69 ± 0.04^{ab}	<0.01
18:3n-3	3.55 ± 0.12^{b}	3.32 ± 0.04^{ab}	3.36 ± 0.04^{ab}	3.23 ± 0.07^{a}	3.16 ± 0.06^{a}	<0.01
20:5n-3 (EPA)	1.14 ± 0.04^{a}	1.47 ± 0.03^{b}	1.88 ± 0.03^{c}	2.82 ± 0.03^{d}	3.37 ± 0.13^{e}	<0.01
22:5n-3 (DPA)	0.88 ± 0.16^{a}	1.01 ± 0.08^{a}	1.38 ± 0.07^{b}	2.35 ± 0.09^{c}	2.84 ± 0.09^{d}	<0.01
22:6n-3 (DHA)	4.56 ± 0.64^{a}	5.25 ± 0.53^{a}	6.12 ± 0.14^{ab}	7.96 ± 0.46^{b}	9.46 ± 0.34^{c}	<0.01
SFA	23.44 ± 0.48^{b}	23.09 ± 0.68^{b}	23.38 ± 0.61^{b}	21.92 ± 0.51^{ab}	21.30 ± 0.36^{a}	0.03
MUFA	35.78 ± 0.58^{c}	36.45 ± 0.42^{c}	36.15 ± 0.47^{c}	33.70 ± 0.37^{b}	31.74 ± 0.28^{a}	<0.01
n-6 PUFA	24.85 ± 0.58^{c}	23.48 ± 0.43^{bc}	23.19 ± 0.24^{ab}	22.31 ± 0.30^{ab}	21.81 ± 0.32^{a}	<0.01
n-3 PUFA	10.99 ± 0.77^{a}	11.71 ± 0.58^{a}	12.27 ± 0.76^{a}	16.98 ± 0.43^{b}	19.39 ± 0.29^{c}	<0.01
n-6/n-3 PUFA	2.26 ± 0.16^{c}	1.94 ± 0.05^{b}	1.78 ± 0.06^{b}	1.29 ± 0.04^{a}	1.12 ± 0.01^{a}	<0.01
n-3 HUFA	5.70 ± 0.67^{a}	6.72 ± 0.54^{a}	6.88 ± 0.75^{a}	10.78 ± 0.45^{b}	12.83 ± 0.32^{c}	<0.01

Values represent means of 3 replicate net cages with 2 fish per cage (n = 3) per treatment. Values in each row without sharing a common letter are significantly different ($P < 0.05$). SFA, saturated fatty acid; MUFA, monounsaturated fatty acid; PUFA, polyunsaturated fatty acid; HUFA, highly unsaturated fatty acids.

Table S5. The *sn*-2 position of fatty acids in muscle triacylglycerols (TAG) of *Trachinotus ovatus* fed different n-3 HUFA levels (% total fatty acids).

Item	Groups					*P* value
	D1	D2	D3	D4	D5	
16:0	33.45 ± 0.35[c]	32.18 ± 0.79[bc]	31.69 ± 0.63[b]	31.69 ± 0.24[b]	30.24 ± 0.24[a]	<0.01
18:0	9.82 ± 0.46[b]	11.3 ± 0.86[ab]	9.69 ± 0.78[ab]	8.92 ± 1.02[ab]	8.28 ± 0.53[a]	0.02
16:1	1.62 ± 0.15	1.28 ± 0.15	1.46 ± 0.10	1.54 ± 0.06	1.76 ± 0.08	<0.01
18:1n-9c	16.09 ± 0.44[bc]	16.66 ± 0.47[c]	14.92 ± 0.42[ab]	13.88 ± 0.34[a]	13.57 ± 0.32[a]	<0.01
18:2n-6	15.22 ± 0.31[c]	12.67 ± 0.60[a]	13.60 ± 0.53[ab]	13.59 ± 0.40[ab]	14.55 ± 0.26[bc]	<0.01
20:4n-6	0.19 ± 0.02[a]	0.83 ± 0.02[b]	0.81 ± 0.02[b]	0.82 ± 0.02[b]	0.82 ± 0.06[b]	<0.01
18:3n-3	1.43 ± 0.08	1.20 ± 0.08	1.32 ± 0.06	1.39 ± 0.05	1.48 ± 0.06	0.04
20:5n-3 (EPA)	0.41 ± 0.03[a]	0.54 ± 0.02[ab]	0.71 ± 0.04[b]	1.05 ± 0.05[c]	1.26 ± 0.08[d]	<0.01
22:5n-3 (DPA)	1.83 ± 0.12[a]	2.80 ± 0.10[b]	2.95 ± 0.07[bc]	3.37 ± 0.12[d]	3.17 ± 0.06[cd]	<0.01
22:6n-3 (DHA)	7.96 ± 0.28[a]	9.36 ± 0.30[b]	11.75 ± 0.47[c]	13.27 ± 0.26[d]	14.80 ± 0.24[e]	<0.01
SFA	45.26 ± 0.57[b]	44.79 ± 1.09[b]	42.85 ± 1.08[ab]	42.14 ± 0.81[a]	40.52 ± 0.58[a]	<0.01
MUFA	18.80 ± 0.52[b]	18.93 ± 0.49[b]	17.28 ± 0.54[a]	16.29 ± 0.36[a]	16.29 ± 0.29[a]	<0.01
n-6 PUFA	15.94 ± 0.31[b]	13.96 ± 0.61[a]	14.89 ± 0.48[ab]	14.78 ± 0.39[ab]	15.70 ± 0.26[b]	<0.01
n-3 PUFA	11.98 ± 0.40[a]	14.18 ± 0.40[b]	16.98 ± 0.59[c]	19.31 ± 0.29[d]	20.91 ± 0.31[e]	<0.01
n-6/n-3 PUFA	1.34 ± 0.06[d]	0.99 ± 0.06[c]	0.88 ± 0.03[b]	0.77 ± 0.02[a]	0.75 ± 0.01[a]	<0.01
n-3 HUFA	8.38 ± 0.30[a]	9.89 ± 0.31[b]	12.47 ± 0.50[c]	14.32 ± 0.25[d]	16.06 ± 0.30[e]	<0.01

Values represent means of 3 replicate net cages with 2 fish per cage (n = 3) per treatment. Values in each row without sharing a common letter are significantly different ($P < 0.05$). SFA, saturated fatty acid; MUFA, monounsaturated fatty acid; PUFA, polyunsaturated fatty acid; HUFA, highly unsaturated fatty acids.

Table S6. Clean data, quality control and summary of Illumina Paired-end sequencing and assembly.

	D2	D5
Raw reads	43959586	45605936
Total raw bases (bp)	6637897486	6886496336
After trimming (clean reads)		
Clean reads	43631590	45273460
Clean bases (bp)	6512274659	6758078327
Error rate(%)	0.0227	0.0225
Q20 (%)[a]	99.01	99.08
Q30 (%)[a]	96.53	96.73
GC content (%)	52.64	52.96
After de novo assembly		
Total sequence base	102235850	
Total unigenes num	126792	
Average length	721.07	
E90N50	1659	
GC percent	46.52	
Mean mapped reads[b]	1844	

[a] Q20 and Q30: the sequencing error rate 0.01 and 0.001 respectively. [b] Mean mapped reads: the average number of clean reads that mapped to each assembly sequence.

Table S7. The result of annotation on unigenes in different databases for *Trachinotus ovatus.*

Database	Annotation	Ration（%）
Swiss-Prot	60585	47.78
NCBI	51624	40.72
Pfam	50516	39.84
KEGG	47171	37.20
GO	17928	14.14
COG	17807	14.04
Total	126792	100.00

NCBI: National Center for Biotechnology Information; KEGG: Kyoto Encyclopedia of Genes and Genomes;GO, Gene Ontology; COG, Clusters of Orthologous Groups.

Disclaimer/Publisher's Note: The statements, opinions and data contained in all publications are solely those of the individual author(s) and contributor(s) and not of MDPI and/or the editor(s). MDPI and/or the editor(s) disclaim responsibility for any injury to people or property resulting from any ideas, methods, instructions or products referred to in the content.
